# Biopsychosocial Measures Related to Chronic Low Back Pain Postural Control in Older Adults

**DOI:** 10.3390/healthcare5040074

**Published:** 2017-10-14

**Authors:** Ryan Hulla, Robert J. Gatchel, Angela Liegey-Dougall

**Affiliations:** Department of Psychology, University of Texas at Arlington, Arlington, TX 76019, USA; ryan.hulla@mavs.uta.edu (R.H.); adougall@uta.edu (A.L.-D.)

**Keywords:** biopsychosocial, older adults, physical, postural control, balance

## Abstract

This study examined the biopsychosocial measures related to postural control in the growing population of older adults (i.e., 60 years and older). The sample of the study consisted of 129 older adults (M = 74.45, SD = 6.95), with 34 males and 95 females; 36 were classified with chronic low-back pain (CLBP), and 93 without chronic low-back pain (NCLBP). Physical and psychosocial constructs were analyzed as predictors for postural control measures. Additionally, gender and classification of low-back pain were examined as moderators for all physical and psychosocial measures. Results demonstrated that physical and psychosocial measures were able to significantly predict composite, visual, and vestibular balance measures, but not somatosensory or preference balance measures. The chair-stand test, modified sit-and-reach test, sleep disturbance, and balance efficacy were all identified as individually significant predictors. Gender and CLBP did not moderate the utility of any predictor variables. Results of the current study re-confirm the importance of utilizing the biopsychosocial approach for future research examining postural control in older adults.

## 1. Overview

In an earlier Special Issue on Healthcare, we highlighted the epidemic of low back pain [1] especially in the elderly [2]. In older adults, such low back pain is often precipitated by slips and falls. Moreover, there is a great deal of biopsychosocial distress associated with slips and falls, such as anxiety of falling, evading activities due to anxiety of falling, and decreased postural control efficacy; these are often taxing phenomena among older adults [3,4,5]. Fall-risk can be intrinsic or extrinsic in nature. Intrinsic factors of fall-risk include advanced age, previous falls, muscle weakness, gait and balance problems, poor vision, postural hypotension, chronic illness such as arthritis, diabetes, stroke, Parkinson’s, and fear of falling. Extrinsic factors include environmental risks such as a lack of handrails, stairs, a lack of bars in the bathroom, dim lighting, slippery and uneven surfaces, psychoactive medications, and improper use of assistive devices [6]. Fall-risk is increased in the older adult population due to psychosocial risk factors, including anxiety and depression [7]. Fear of falling is defined as an unrealistic preoccupation about falling or the belief that the individual cannot prevent or avoid a fall. An intense anxiety of falling can be experienced by older adults that have or have not fallen. Awareness of the severity of falls may instill a fear in individuals, leading to behavior changes that limit functional capacity [8]. In contrast, there are beneficial health advantages that older adults may experience with increased physical activity [9]. With the population of older adults increasing in the United States at a rapid pace, the country has entered what is sometimes referred to as a “longevity revolution” or “graying of America”. This increase can be a catalyst of healthcare economic stress due to physical and psychosocial vulnerabilities of elderly. Older adults are at higher risk of sustaining physical injury and psychosocial trauma from a slip or fall [10]. This will be reviewed next.

As a start, the 2010 U.S. census reported that, from the 2000 through 2010 decade, the elderly population in America (65 years and older specifically) increased exponentially more (15.1%) than the total U.S population (9.7%).The population of Americans 65 years or older was recorded 40.3 million in April of 2010 [11]. In the year of 2014, 15% of Americans were 65 years or older. In 2030 that number is expected to increase to 20% and, by 2060, it is projected that 24% of Americans will be 65 years or older [12,13,14,15]. Falls and fall-related injuries: can lead to extensive hospital visits; are one of the principle reasons of diminished health and function in community-dwelling older adults; can be a precursor to physical impairment, pain, chronic pain, fractures, disability, lower-quality-of-life; and, in more extreme cases, are a significant cause of injury-related death in the elderly population [16,17,18]. The elderly population is more vulnerable to falls because of age-associated ailments that include declining vision, absence of physical activity, weakening neuromuscular factors, cognitive deficiencies, osteoarthritis, and the use of diuretic and psychotropic medications [19]. These ailments result in roughly one-fourth of elderly adults falling every year, with one-fifth of them needing medical attention and resulting in serious injury [20]. Older adults experiencing a previous fall are also twice as likely to experience another fall in contrast to their counterparts without a previous fall [21,22,23,24,25]. Annually, one in three older adults over the age of 65 fall, and 50% over the age of 80, will sustain a fall each year [26,27]. In a study conducted by Hageman and colleagues, older adults had greater sway in balance measures when compared to younger adults, but gender did not have a significant effect on balance [28]. A study by Wolfson and colleagues of older adult females had worse postural sway (balance) than older adult males when taking measurements with eyes closed, but no significant differences were observed between genders when taking measurements in eyes-open conditions [29].

In addition, 20–30% of individuals who fall suffer an injury (e.g., low back pain) that will reduce mobility and independence, and increase the risk of premature death [30]. If older adults experience a fall, they can be even at higher-risk for a fall in the future. It has been supported that when physical activity becomes limited, and is disproportionate to an individual’s physical capabilities, sensorimotor deconditioning, reduced postural control, decreased muscular strength, decreased muscular flexibility, and decreased muscular and cardiovascular endurance and increased risk-of-falls can be a consequence from avoidance of activity [31,32,33,34]. Indeed, falls are the leading cause of injury deaths among individuals over the age of 65; and half of the falls will occur in their own home [35,36]. Prior studies have also demonstrated how range-of-motion in the hamstrings can increase with improved postural control [37], and that agility is a reliable and valid predictor of fall risk among adults [38]. Even though psychosocial concerns are predominant in individuals who have sustained a fall, people who have had no history of a previous fall can also be negatively affected. For example, Deshpande and colleagues, in a large population-based investigation involving 926 elderly individuals, found that 70% of individuals who reported having a fear of falling had no history of falls within the prior year [3]. Consequently, the effects of restrictive movement is not just physical in nature in terms of physical deconditioning, but studies support that avoiding activities due to fall-related anxiety can exacerbate social isolation, diminish quality-of-life, and is associated with depressive symptoms [33,39,40].

Within the scientific literature, an array of psychosocial factors associated to falls have been acknowledged, and usually include factors such as anxiety from fear of falling, avoiding activities due to fear of falling, a decline in balance efficacy, and an unrealistic fear about the consequences of falling [34,40,41]. The psychosocial occurrence of having a persistent fear of falling could contribute to exacerbating the threat for later falls, thus further hampering physical activity [39], as well as being an initiator for deviations in gait mechanics that follow disorganized gait features (e.g., exaggerated shorter strides [42]. This further intensifies the threat of a fall. For example, Petrella and colleagues found that the focused-avoidance of physical activity was associated in those who had a fear of falling [43]. Relatedly, a decrease in muscle mass (a marker of physical frailty, often from inactivity) is often seen in older adults with chronic low back pain (CLBP) [44]. Physical activity, in contrast, improves physiological measures, such as muscle tone, balance, and flexibility, making the possibility of experiencing a fall less likely [45].

Older adults often avoid physical activity, making their risk for a fall greater [46]. Not only does declining physical function influence postural control, but evidence indicates that decreased physical function has an impact on psychosocial health [47]. Evasion from physical movement adversely affects an older adult’s postural control, and also diminishes the quality of mental well-being. For example, research conducted by Morgan and colleagues found a strong relation between physical activity and psychological well-being, demonstrating that older adults who are consistent in being physically active are less susceptible to suffer depression, and have improved psychological well-being [48]. Balance efficacy can be defined as a person’s perceived self-confidence regarding his/her ability to avoid falls while performing identified activities [26]. Balance efficacy has been supported to be a predictor of: sustained falls in individuals [49], poor postural control, reduced physical function, and increased fall risk [50]. Klima and colleagues found that balance efficacy was significantly correlated with balance-ability and functional mobility in older adult men [51]. A lack of physical activity, or living a sedentary lifestyle, leads to a decline in postural control and, therefore, increased fall risk [47]. Adequate exercise can have an influence in reducing falls in older adults by enhancing biopsychosocial functioning [47]. 

The relationship among psychosocial constructs (i.e., anxiety, depression, and sleep disturbance), low-back pain, and falls is not comprehensively understood. Therefore, research still needs to delineate the associations among psychosocial measures, CLBP, recent falls sustained, fall-risk, and exercise intervention efficacy to positively impact these aforementioned variables. To date, pain has been documented to have a relationship with mobility restrictions [52], as well as balance and gait deficits [53,54,55]. An earlier study conducted by Hulla and colleagues provides an example of these new important issues [2]. The study found that, in demographically-matched elderly individuals, those with CLBP were significantly worse on constructs of physical function, fatigue, pain interference (and approached significance for sleep disturbance). It was also found that NeuroCom balance measures (a force plate that measures center of gravity in several different conditions in which the surrounding wall and a force plate move in relation to the sway of the participant), and psychosocial measures significantly predicted CLBP.

In another study, Stubbs and colleagues found that, in the community-dwelling older population, pain was a significant predictor in “fall efficacy” or an individual’s perception of confidence in avoiding a fall, balance confidence, fear of falling, and perceived consequences of falling [56]. Older adults are also more vulnerable to experience psychosocial ailments because of their susceptibility to pain disorders (e.g., Bradbeer and colleagues found that older adults who demonstrated signs of depression were more likely to have symptoms of chronic pain than their counterparts who are not depressed) [57]. Not unlike depression, anxiety can influence the experience of chronic pain [58]. The older-adult population tends to have greater anxiety, and this can be evidenced in regards to fear of falling [59]. This fear can lead to limited [58] which can, in turn, intensify pain and weaken balance capabilities. A general explanation of the compounding problem with chronic pain and aging affecting psychosocial well-being is well-supported by a body of evidence. For example, in cases of chronic osteoarthritis, pain was associated with physical inactivity, which is then followed by further avoiding physical activity [60] that intensifies the series of reduced participation in physical activity and reduced physical function, and these intensifying aliments of sarcopenia [42], osteoporosis [16], and stiffening of skeletal muscles.

Pain, also has been shown to influence sleep quality [61]. Sleep quality influence balance capabilities. For example, a study conducted by Furtado and colleagues demonstrated the individuals with worse sleep quality performed worse on balance test with eyes closed [62]. Jorgensen and colleagues found that participants who described being “sleepy” had increased postural sway, relative to participants who reported being well-rested [63]. Other research has shown that sleep disorders have a negative impact on postural control and balance deterioration that are responsible for work accidents and falls among older adults, and can occur just after one night without sleep [64,65,66,67,68,69,70,71,72,73,74]. 

Identifying objective variables related with fall-risk would be beneficial in order to diminish an individual’s threat of sustaining a fall. Because experiencing a fall can be a “shattering experience” on the mental well-being, physical functioning, and overall quality-of-life in older adults, it is crucial that convenient, economical, and easy administrable tests are accessible for routine clinical use [75]. As an initial step in this progression of these methods, the present study evaluated what biopsychosocial variables were related to postural control in older adults. The current study is innovative because very few studies have examined biopsychosocial measures in association with the multiple components involved in balance.

## 2. Materials and Methods 

### 2.1. Participants

Participants consisted of older adults (age 60 years and older) from the Center for Healthy Living and Longevity (CHLL), who had participated in the CHLL program from August 2015 through May 2017 at the University of Texas at Arlington. All measurements were taken prior to participation in the CHLL program each semester. The study focused on elderly adults (60 years and older), recruited from the local community through scheduled presentations about the CHLL at various places, including retired faculty gatherings, word-of-mouth from friends, churches, and doctor recommendations. All participants had provided informed consent to participate per the Institutional Review Board (IRB) at the University of Texas at Arlington. The sample demographics included 34 males and 95 females; 36 participants had CLBP, and 93 participants did not (NCLBP). The mean of the participants was age 74.45 years, with a standard deviation of 6.95 years. 

### 2.2. Instruments and Procedures

The PROMIS-29 is a computer-adaptive test, designed to measure the following seven psychosocial constructs: physical function; anxiety; depression; fatigue; sleep disturbance; ability to participate in social roles and activities; and pain interference. The PROMIS-29 has been tested and validated for concurrent and discriminant validity, test-retest reliability, as well as participant preference for measuring health-related quality-of-life. Each participant in the present study was assigned a computer, and a test profile was created before taking the assessment. The NIH case-definition of CLBP was used to classify participants as having or not having CLBP. The criteria for having CLBP included: having low-back pain for greater than three months; and having low-back pain at a frequency of at least half the days in the past six months. 

The NeuroCom Balance System detects any changes in an individual’s balance over time by evaluating their aptitude to regulate their center of gravity in various motor and sensory conditions. The participant stood on a force plate, facing into a three-sided surround. The visual surround and force plate moved in reaction to the participants’ forwards and backwards sway, generating a distorted proprioceptive or visual input to the brain. Postural control was measured by using the sensory organization test (SOT) and strategy analysis under six conditions, with a NeuroCom Balance System. Throughout the assessment, inaccurate information was delivered to the participant’s eyes, feet, and joints through “sway referencing” of the support surface and/or the visual surround. The participants were fitted with a cushioned vest that was attached to the NeuroCom Balance System outer-structure, in order to prevent the participant from an actual fall. Each condition was executed three times. Outcome measures for this test included: (1) strategy analysis, which measures the relative amount of movement of ankle-strategy and hip-strategy the participant used to maintain balance throughout each trial; (2) equilibrium score, which quantifies the center of gravity (COG) sway or postural stability; (3) sensory analysis ratios, which were used in conjunction with the participant’s equilibrium scores to detect deficiencies of the participant’s sensory systems; and (4) COG alignment, which plots the individual’s COG position at the beginning of each trial of the SOT, in which each mark determines COG alignment during a single SOT trial, relative to the center of the base of support. The four measures was then combined for use as a composite score. Along with a composite score, the NeuroCom Balance System also provides separate scores for the somatosensory, visual, and vestibular systems, as well as preference (hip-to-ankle dominance).

After the participants’ postural control was assessed with the NeuroCom Balance System, they were assessed for the components of physical fitness in upper- and lower-limb muscular strength, endurance and flexibility, and cardiovascular endurance through the Senior Fitness Test which evaluates functional fitness performance of elderly adults [76]. Participants were then questioned about the amount of falls they had in the past year and six months. For the current study purposes, the physical measures examined from the Senior Fitness Test were lower-body measures in nature, and included: chair stands completed in 30-s; a 6-min walk test that measures how far the participant can walk in 6-min; an eight feet-get-up-and-go agility test (which consists of the participants standing up out of a chair and moving around a marker that is eight feet from the chair, and then sitting back down); and a modified sit-and-reach test to measure lower body flexibility.

Participants were first consented to the IRB-approved protocol of the current study. After consent, participants filled out the NIH case-definition of CLBP inventory, with paper and pencil. The Patient Reported Outcome Measurement Information System Computer Adaptive Test (PROMIS) was then administered, followed by a validated demographic information risk assessment survey (the Comprehensive Fall Risk Screening Instrument (CFRSI) [77]. The CFRSI included five subscales, including the American Geriatric Society [78] fall-risk factors that included history of falls, the use of an assistive device, arthritic diagnosis, and age of an individual. The physical risk subscale includes a mobility test and an agility/speed test score that was also measured in the SFT. A medication subscale also included information regarding the use of high-risk medication (i.e., psychotropic, anti-arrhythmic, digoxin/lanoxin, and diuretics), information of multiple pharmacists, and the experience of medication side-effects. The vision subscale included visual acuity, visits to the optometrists, and the use of prescription lenses. The environment subscale was calculated with information regarding hazards that exist in the participants’ home. The relative risk ratio and odds ratio were used to weigh each, and to calculate each fall-risk subscale. All five of the subscale scores were averaged for a total fall-risk score [77]. The Balance Efficacy Scale (BES), which is an 18-question psychometric assessment of balance confidence survey developed by the California State University at Fullerton’s Center for Successful Aging, was also administered [79]. When the participants finished the PROMIS-29 CAT, they logged-out of their profile, and the results were saved, to be accessed later in order to be de-identified, and transferred and coded into IBM’s Statistical Package for Social Science (SPSS IBM Corporation, Armonk, New York, U.S.A.). 

After the PROMIS-29 was administered, participants took the SOT on the NeuroCom Balance System to acquire postural control scores. The last test administered was the Senior Fitness Test. All measures were collected on a score sheet, and then again de-identified and inputted into SPSS for statistical analysis. All tests were administered by a senior graduate student, and graduate and undergraduate research assistants.

In summary, the current study examined the following biopsychosocial variables: physical function, sleep disturbance, fatigue, pain interference, depression, and anxiety, as measured by the (PROMIS); balance efficacy as measured by the BES; and physical measures (cardiovascular endurance (six-minute walk test), lower body muscular endurance (chair-stand test), lower body flexibility (modified and sit-and-reach test), and agility (eight feet-get-up-and-go test)). It also examined if CLBP and gender moderated the utility of any of the aforementioned biopsychosocial predictors, while controlling for age (self-report), calculated environmental fall-risk, and medication fall-risk measured by the subscales of the CSFRI. The assumption was that the aforementioned biopsychosocial measures would predict overall NeuroCom composite, visual, vestibular, somatosensory, and preference scores of the SOT, as measured by the NeuroCom Balance System (a computerized dynamic posturography device).

### 2.3. Power Analysis

A priori multiple regression G*Power [80], with 12 predictor variables requiring a medium effect size of 0.15, and a regression critical coefficient of 1.84, indicated that a sample size of N = 127, was needed to find significance at a probability error of α = 0.05. With a sample size of N = 129, the study was sufficiently powered to use up to 12 predictors in the regression models.

### 2.4. Statistical Analysis for Psychosocial Predictors

A principal components analysis was conducted on psychosocial and physical variables separately to use as covariates in the multiple hierarchical multiple regression models that examined psychosocial and physical measures separately as the predictor variables, while maintaining a proper power for the analyses. Using multiple hierarchical multiple regression models, composite, visual, vestibular, somatosensory, and preference balance scores were the dependent variables for each of the hierarchical multiple regression models. Physical measures from the Senior Fitness Test, were calculated into a factor score coefficient (FSC), with a principal component analysis that was used as a covariate in order to measure the predictability of the psychosocial variables, while controlling for physical measures and maintaining the proper amount of predictors for the models’ power. Along with the FSC of the physical measures, age, calculated environmental fall risk, and calculated medication fall risk were covariates in the analysis. In the first step of each hierarchical multiple regression model (five separate models to measure the predictability of composite, visual, vestibular, somatosensory, and preference balance scores), the covariates were entered into step one of the model to measure. In step two of each hierarchical multiple regression model, the six psychosocial predictor variables (anxiety, depression, fatigue, pain interference, sleep disturbance, and balance efficacy) were introduced. For step three of each hierarchical multiple regression model, the moderators of CLBP and gender were introduced. Finally, in step four of each hierarchical multiple regression model, the interaction between the moderators and predictor variables were introduced.

### 2.5. Statistical Analysis for Physical Predictors

Using multiple hierarchical multiple regression models, visual, vestibular, somatosensory, and preference balance scores, were the sole dependent variables for each hierarchical multiple regression model. Psychosocial measures of anxiety, depression, fatigue, sleep disturbance, pain interference, and balance efficacy (as measure by the PROMIS and BES, respectively), were calculated into a FSC with a principal component analysis that was used as a covariate in order to measure the predictability of the physical variables, while controlling for psychosocial measures and maintaining the proper amount of predictors for the models’ power. Along with the FSC of the psychosocial measures, age, calculated environmental fall risk, and calculated medication fall risk were covariates in the analysis. In the first step of each hierarchical multiple regression model (five separate models to measure the predictability of composite, visual, vestibular, somatosensory, and preference balance scores), the covariates were entered into step one of the model to measure. In step two of each hierarchical multiple regression model, the four physical predictor variables (modified sit-and-reach, chair stands, eight feet-get-up-and-go, and six minute walk) were introduced. For step three of each hierarchical multiple regression model, the moderators of CLBP and gender were introduced. Finally, in step four of each hierarchical multiple regression model, the interaction among the moderators and predictor variables were introduced.

### 2.6. Ethical Statement 

All subjects gave their informed consent for inclusion before they participated in the study. The study was conducted in accordance with the Declaration of Helsinki, and the protocol was approved by the Institutional Review Board (IRB) of the University of Texas at Arlington (IRB number: 2016-0117.6).

## 3. Results

### 3.1. Factor Analyses

For the models containing physical measures as the predictor variables, the factorability of the six psychosocial items were initially examined. The Kaiser-Meyer-Olkin measure of sampling adequacy was 0.76, above the recommended value of 0.60, and Bartlett’s test of sphericity was significant (χ^2^ (21) = 250.84, *p* < 0.001). Given the overall indicators, a factor analysis was conducted with all six psychosocial constructs. A principal components analysis was used because the purpose was to compute a composite psychosocial score to use as a covariate in the multiple hierarchical multiple regression models that examined physical measures as the predictor variables, while maintaining proper power for the analyses. The initial eigenvalues showed that the first factor explained 44.98% of the variance, and the second factor explained 16.48% of the variance. All psychosocial constructs were most heavily loaded on the first factor: anxiety (*r* = 0.65), depression (*r* = 0.65), fatigue (*r* = 0.82), pain interference (*r* = 0.70), physical function (*r* = 0.71), sleep disturbance (*r* = 0.45), and balance efficacy (*r* = 0.66). Since all constructs were most heavily weighted on the first factor, the first factor coefficient was used as the covariate in the multiple hierarchical regression models that examined the physical measures as the predictor variables. For the models containing psychosocial measures as the predictor variables, the factorability of the four physical measures were initially examined. The Kaiser-Meyer-Olkin measure of sampling adequacy was 0.75, above the recommended value of 0.60, and Bartlett’s test of sphericity was significant (χ^2^ (21) = 145.27, *p* < 0.001). Given the overall indicators, a factor analysis was conducted with all four physical measurements. The initial eigenvalues showed that the first factor explained 61.44% of the variance. All psychosocial constructs were most heavily loaded in the first and only factor produced: eight feet-get-up-and-go (*r* = 0.88); six minute walk (*r* = 0.81); modified sit-and-reach (*r* = 0.59); and chair stands (*r* = 0.83. Since all constructs were most heavily weighted on the first factor, the first factor coefficient was used as the covariate in the multiple hierarchical regression models that examined psychosocial constructs as the predictor variables.

### 3.2. Multiple Hierarchical Regression Analyses

Results of the hierarchical multiple regressions for the covariates (i.e., psychosocial variables, age, medication use, and environmental risk) in the first step did not predict composite, visual, vestibular, somotasensory, or preference balance scores. In the second step, physical measures (i.e., sit-and-reach test, chair stands test, eight feet-get-up-and-go test, six minute walk test) were significant variables after accounting for the covariates for predicting NeuroCom composite (*p* < 0.001; ΔR^2^ = 0.25, *p* < 0.001) and vestibular balance scores (*p* = 0.008; ΔR^2^ = 0.19, *p* = 0.001). Physical measures did not yield statistically significant results for predicting the NeuroCom somatosensory or preference scores. The physical measures that were significant variables for predicting composite balance scores were the sit-and-reach measure (*p* = 0.035, sr^2^ = 0.22) and the chair stand measure (*p* = 0.001, sr^2^ = 0.35). The 6-min walk and eight feet-get-up-and-go test were not found to be statistically significant as individual predictors for composite balance scores. All physical predictor variables were also analyzed if they were moderated by gender or CLBP. No interactions were significant in predicting composite balance scores. In regard to physical measures that were significant individual predictors for vestibular scores, these were: sit-and-reach measure (*p* = 0.037, sr^2^ = 0.22) and the chair stand measure (*p* = 0.014, sr^2^ = 0.26). The 6-min walk and eight feet-get-up-and-go test were not found to be statistically significant as individual predictors for the vestibular balance scores. All physical predictor variables, as in the previous model, were analyzed to examine if they are were moderated by gender or CLBP. No interactions were significant when predicting vestibular balance scores. All tolerance (>0.01) and variance inflation factors (VIF) (<10.00) measures were within acceptable limits, indicating no issues with multicollinearity in any of the analyzed models. Results of the hierarchical multiple regressions for the covariates in the first step did significantly predict composite (R^2^ = 0.19, *p* < 0.001) and vestibular (R^2^ = 0.12, *p* = 0.017) balance scores in the first step, but did not predict visual, somatosensory, or preference balance scores. In the second step of the hierarchical multiple regression model, psychosocial measures were introduced (i.e., anxiety, depression, fatigue, pain interference, physical function, sleep disturbance, and balance efficacy), and when controlling for covariates in the first step of the regression model. The psychosocial measures yielded significant results after accounting for the covariates for predicting the NeuroCom composite (*p* < 0.001; ΔR^2^ = 0.14, *p* = 0.018), and visual balance scores (*p* = 0.017; ΔR^2^ = 0.15, *p* = 0.031). Psychosocial variables did not yield statistically significant results for predicting the NeuroCom somatosensory or preference scores. The psychosocial measures that were significant variables for predicting composite balance scores were: sleep disturbance (*p* < 0.001, sr^2^ = −0.38), and balance efficacy approached significance (*p* = 0.072, sr^2^ = −0.19). Anxiety, depression, fatigue, pain interference, and physical function were not found to be statistically significant as individual predictors. In regard to visual balance scores, significant individual predictors were also sleep disturbance (*p* = 0.001, sr^2^ = −0.34) and balance efficacy (*p* = 0.037, sr^2^ = −0.22). All psychosocial predictor variables were analyzed if they were moderated by gender or CLBP in predicting visual balance scores, but no interactions were statistically significant in predicting visual balance scores. All tolerance (>0.01) and VIF (<10.00) measures were within acceptable limits, indicating no issues with multicollinearity. Descriptive NeuroCom data compared to low and high groups of each significant predictor (i.e., sit and reach, chair stands, sleep disturbance, and balance efficacy) are presented in Table 1, Table 2, Table 3 and Table 4.

## 4. Discussion

The purpose of the current study was to examine the capability of biopsychosocial measures in predicting postural control. Results of the current study physical measures significantly predict composite balance scores and vestibular balance scores. Physical measures did not significantly predict somatosensory balance scores or preference balance scores, the models that were significant for both composite and vestibular balance scores. The physical variables of chair stands and the modified sit-and-reach were, individually, significant predictors in both models. In the case of the psychosocial measures, they were significant in being able to predict composite and visual balance scores. The models that were significant for composite and visual balances, sleep disturbance, and balance efficacy were significant individual predictors in both models.

The results also demonstrated that the chair-stand test was a significant individual predictor of balance scores. This supports the previous findings of Granacher and colleagues, in which postural control was found to coincide with an increased rate-of-force development in lower body extensor muscles [81]. The Hopper and colleagues study of inducing lower-body muscular fatigue through 60-s of repetitive jumping also supported the results of the current study [82]. It could be that the neuromuscular adaptations that occur in muscle strength gains in the lower body may be influencing a participant’s ability in postural control. It is well known that muscular responses to training are adapted and maintained by specific changes occurring within the neural control circuits [83]. In normal conditions strength training may elicit adaptations along the neuromuscular chain from the higher brain centers down to individual muscle fibers. This neural drive is thought to increase in agonist muscle recruitment, neural firing rate, enhanced motor neuron synchronization during muscular contractions, increased length of nerve terminal branching, increased motor neuron end-plate perimeter length, and a reduction in inhibitory mechanisms from the Golgi tendon organs that may occur [83,84,85,86]. A study by Adams and colleagues found, using magnetic resonance imaging, that only 71% of muscle tissue was activated during maximal effort in untrained individuals [87], and it has been found that training can greatly reduce this deficit [88]. A systematic review of balance training on neuromuscular control by Zech and colleagues found that balance training may allow increased muscular activity for the lower-body muscles of the soleus, gastrocnemius medialis, and quadriceps muscles [89]. The enhancement in the neuromuscular system could be why participants who performed more repetitions on the chair-stand test were predicted to have better overall composite, vestibular, and visual balance scores in the current study.

Previous studies have found musculoskeletal tightness and muscle strains to influence muscular strength [90,91]. In this study, results of the chair-stand test and the modified sit-and-reach test were both individually significant predictors among balance scores. The results of this study also support Taylor-Piliae and colleagues implementation of a Tai chi exercise program over a 12-week period, which enhanced balance, strength, and flexibility measures [92].

Sleep disturbance was a significant individual predictor for balance scores. This supports the findings of Jorgensen and colleagues, who found that sleep-deprived participants had increased postural sway versus participants who reported being well-rested [63]. Furtado and colleagues also demonstrated that individuals with decreased sleep-quality perform worse on a balance test with eyes closed [62]. Sleep disorders had a negative impact on the postural control and balance deterioration that are responsible for work accidents and falls among older adults, and can occur just after one night without sleep [64,65,66,67,68,69,70,71,72,73,74]). A study of older adult participating in Tai chi classes for six months found that, compared to controls, those who participated in these Tai chi classes had a significantly lower fear of falling, and improved sleep quality [93], thus supporting the findings of the current study. Indeed, lower sleep quality in older adults appear to be significantly related to impaired health status, decreased physical activity, low level of physical functioning, and an increase prevalence of chronic diseases [94,95]. Alternative exercise, such as Tai chi, have been found to improve sleep quality, sleep-onset latency, sleep duration, sleep efficiency, and sleep disturbance [96]. The participants in this study were more generally more active. 

Balance efficacy was also an individually significant predictor of balance scores. For the psychosocial constructs associated with falls some of the most common factors are increased anxiety from fear of falling, and a reduction in balance efficacy [33,39,40]. In this study, balance efficacy was a significant predictor for balance scores. However, anxiety was not found to be a significant predictor.

It should be noted that, in any clinical research study of this type, a potential for some confounding elements may or may not have influenced the results. For example, testing procedures were carried out by numerous graduate and undergraduate research assistants and, even though all research assistants were trained before any data collection, the research assistants could have influenced the effort or performance of the participant based on gender, familiarity with the research assistant, or strictness in the testing protocol. Additionally, selection of participants was not randomized, but were volunteers who had an interest in participating in the current study.

## 5. Conclusions

Overall, the major aims of the present study embraced a biopsychosocial model. Indeed, there is a need to not just consider physical measures when assessing postural control, but to simultaneously evaluate psychosocial constructs with physical measures when examining fall-risk in older adults. A major innovation of this investigation was the use of a relatively new physical measure of postural control, and the examination of its relationship to other physical, psychosocial, and pain measures in an older adult population.

## Figures and Tables

**Table 1:** Neurocom data descriptives compared with low and high balance efficacy.

Measure	Low BE (*n* = 47)			High BE (*n* = 77)		
	M	SD	Range	M	SD	Range
Composite Balance	72.85	8.26	48.00–84.00	73.96	7.16	54.00–89.00
Visual	85.94	8.29	63.00–101.00	86.3	9.34	51.00–105.00
Vestibular	64.60	17.90	4.00–88.00	64.97	13.03	17.00–89.00
Somatosensory	93.55	6.14	68.00–102.00	94.88	3.90	85.00–107.00
Preference	99.43	12.55	72.00–152.00	97.31	8.18	80.00–117.00

Chair stands is represented by CS. Low and high groups were divided with a median split.

**Table 2:** Neurocom data descriptives compared with low and high sleep disturbance.

Measure	Low SLD (*n* = 67)			High SLD (*n* = 56)		
	M	SD	Range	M	SD	Range
Composite Balance	75.19	6.39	58.00–87.00	72.05	8.70	48.00–89.00
Visual	87.66	7.83	63.00–101.00	84.69	10.11	51.00–105.00
Vestibular	67.19	12.27	13.00–89.00	62.54	17.68	4.00–85.00
Somatosensory	94.21	4.04	87.00–103.00	94.69	5.81	68.00–107.00
Preference	99.64	10.00	82.00–152.00	97.21	9.05	78.00–115.00

Chair stands is represented by CS. Low and high groups were divided with a median split.

**Table 3:** Neurocom data descriptives compared with low and high sit and reach scores.

Measure	Low SNR (*n* = 44)			High SNR (*n* = 85)		
	M	SD	Range	M	SD	Range
Composite Balance	72.25	7.95	48.00–84.00	74.48	7.31	50.00–89.00
Visual	86.32	9.02	65.00–105.00	86.40	8.90	51.00–100.00
Vestibular	63.02	18.98	4.00–88.00	66.18	12.22	18.00–89.00
Somatosensory	93.11	6.14	68.00–107.00	95.11	3.90	87.00–103.00
Preference	100.48	11.70	78.00–152.00	97.01	8.69	71.00–117.00

Chair stands is represented by CS. Low and high groups were divided with a median split.

**Table 4:** Neurocom data descriptives compared with low and high chair stand scores.

Measure	Low CS (*n* = 66)			High CS (*n* = 54)		
	M	SD	Range	M	SD	Range
Composite Balance	71.14	7.01	48.00–85.00	77.59	6.14	62.00–89.00
Visual	84.33	9.45	51.00–101.00	89.87	7.06	63.00–105.00
Vestibular	60.70	16.10	4.00–89.00	70.90	9.89	41.00–88.00
Somatosensory	93.91	4.00	85.00–102.00	95.54	4.40	85.00–107.00
Preference	97.53	11.79	72.00–152.00	99.26	7.40	84.00–119.00

Chair stands is represented by CS. Low and high groups were divided with a median split.
